# The Dog That Didn’t Bark: A New Interpretation of Hypsoporphyrin Spectra and the Question of Hypsocorroles

**DOI:** 10.1021/acs.jpca.1c08425

**Published:** 2021-11-11

**Authors:** Abhik Ghosh, Jeanet Conradie

**Affiliations:** † Department of Chemistry, UiT—The Arctic University of Norway, Tromsø N-9037, Norway; ‡ Department of Chemistry, University of the Free State, P.O. Box 339, Bloemfontein 9300, Republic of South Africa

## Abstract

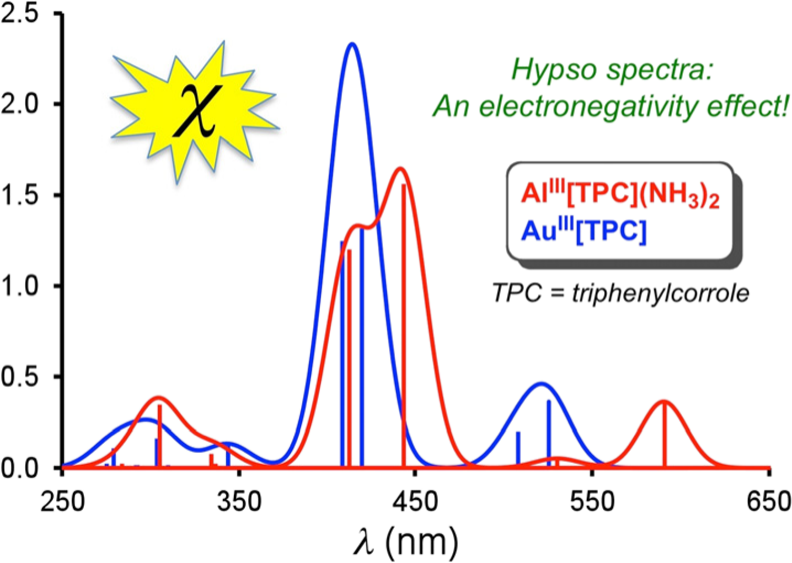

Nearly a half-century after Gouterman classified the UV–vis–NIR spectra of porphyrin derivatives as normal, hyper, or hypso, we propose a heretofore unsuspected “mechanism” underlying hypso spectra. Hypsoporphyrins, which exhibit blueshifted optical spectra relative to normal porphyrins (such as Zn porphyrins), typically involve d^*n*^ transition metal ions, where *n* > 6. The spectral blueshifts have been traditionally ascribed to elevated porphyrin e_g_ LUMO (lowest unoccupied molecular orbital) energy levels as a result of antibonding interactions with metal d_π_ orbitals. Herein, we have found instead that the blueshifts reflect a lowering of the a_2u_ HOMO (highest occupied molecular orbital) energy levels. Electronegative metals such as Pd and Pt transfer smaller quantities of electron density to the porphyrin nitrogens, compared to a more electropositive metal such as Zn. With large amplitudes at the porphyrin nitrogens, the a_2u_ HOMOs of Pd(II) and Pt(II) porphyrins accordingly exhibit lower orbital energies than those of Zn(II) porphyrins, thus explaining the hypso effect. Hypso spectra are also observed for corroles: compared with six-coordinate Al(III) corroles, which may be thought of exhibiting normal spectra, Au(III) corroles, for instance, exhibit blueshifted or hypso spectra.

## 1 Introduction

The famous four-orbital model,^1,2^ which explained the electronic absorption spectra of simple porphyrins, was devised by Gouterman in the early 1960s, while he was an Assistant Professor at Harvard. According to this model, the two highest occupied molecular orbitals (HOMOs) (a_1u_ and a_2u_ under *D*_4h_ symmetry) and the two lowest unoccupied molecular orbitals (LUMOs) (e_g_) are energetically well-separated from all other occupied and virtual molecular orbitals (MOs). The Q and Soret bands are then explained by transitions between these four MOs, taking configuration interaction into account. Some 15 years later, now on West Coast, he presented an optical taxonomy of porphyrins in a lengthy chapter in Dolphin’s multivolume work*The Porphyrins*.^3^ He classified porphyrins into three major classes—normal, hypso, and hyper. Normal porphyrins exhibit electronic absorption spectra that can be largely accounted for with the four-orbital model. Hypsoporphyrins exhibit blueshifted spectra, typical examples including d^*n*^ metalloporphyrins for *n* > 6. In contrast, hyperporphyrins exhibit redshifted optical spectra and/or extra absorption bands above 300 nm. Typical examples include d^*n*^ metalloporphyrins with *n* < 6, which in turn include many heme proteins and their intermediates and model compounds. Substituents and other structural perturbations can also lead to hyper spectra.^4^

Many, but not all, hypsoporphyrins, especially the noble metal porphyrins, are moderately to strongly phosphorescent.^5−7^ Their long-lived triplet states have been exploited for oxygen sensing and photodynamic therapy.^8−12^ Gouterman and co-workers famously exploited platinum(II) porphyrins to devise pressure-sensitive paints for airplane wings.^13−16^ More recently, 5d metallocorroles,^17−21^ including ReO,^22−24^ OsN,^25,26^ Ir,^27−29^ Pt,^30,31^ and Au^20,32−35^ corroles, have been found to exhibit NIR phosphorescence under ambient conditions, raising the question whether they, or at least some of them, should be described as hypsocorroles.

**Figure 1:**
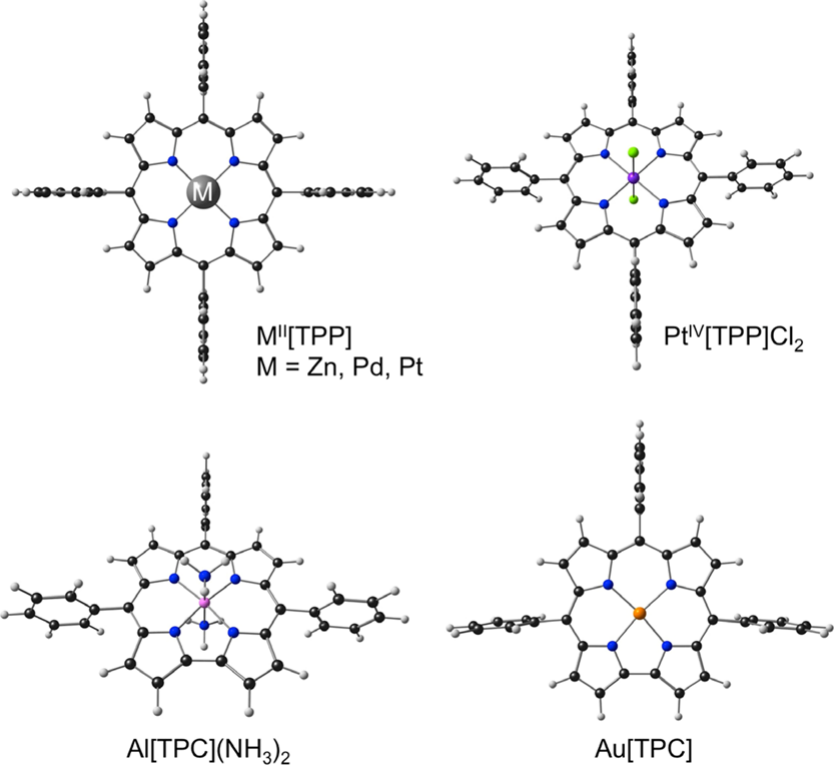
Molecules studied in this work.

Remarkably, in spite of their broad importance, few hypso and hyper spectra have been examined by means of modern quantum chemical methods, such as time-dependent density functional theory (TDDFT) calculations,^36−38^ which prompted us to undertake a first such investigation of selected hypsoporphyrin systems. Thus, we examined several M^II^[TPP] derivatives (where M = Zn,^39,40^ Pd,^41^ and Pt^41^), Pt^IV^[TPP]Cl_2_,^42^ and two corroles, Al^III^[TPC](NH_3_)_2_^43^ and Au^III^[TPC]^34^ (Figure 1). Of these, only Pd^II^[TPP] and Pt^II^[TPP] are clearly hypsoporphyrins, while Au[TPC] is a potential hypsocorrole. The other complexes are included for comparison.

The hypso effect has traditionally been explained in terms of metal(d_π_)–porphyrin(LUMO) orbital interactions.^3^ By engaging in backbonding interactions with the porphyrin e_g_ LUMOs, the d_*xz*_ and d_*yz*_ orbitals are stabilized. The corresponding antibonding MOs, that is, the LUMOs, the theory goes, are destabilized, which results in an elevated HOMO–LUMO gap, explaining the hypsochromic shifts of the Q and Soret bands. To our surprise, the present reinvestigation provided no support whatsoever for this long-held picture, suggesting instead an entirely different “mechanism” underlying hypso spectra.

## 2 Computational Methods

All calculations were carried out with the ADF^44^ 2018 program with all-electron ZORA-STO-TZ2P basis sets, fine meshes for numerical integration of matrix elements, and adequately tight convergence criteria for both SCF and geometry optimization cycles. Molecular geometries were optimized with OLYP^45,46^-D3,^47^ with *D*_4h_ and *C*_2v_ symmetry constraints for the porphyrin and corrole derivatives, respectively. These optimized geometries were then used for TDDFT calculations with the OLYP-D3, B3LYP* (15% exact exchange), and CAMY-B3LYP^48−50^ functionals. B3LYP^51,52^-D3-optimized geometries were used for the TDDFT calculations with the B3LYP functional. The COSMO^53^ solvation model (with dichloromethane as the solvent) was used throughout.

## 3 Results and Discussion

### 3.1 Theoretical Model

**Figure 2:**
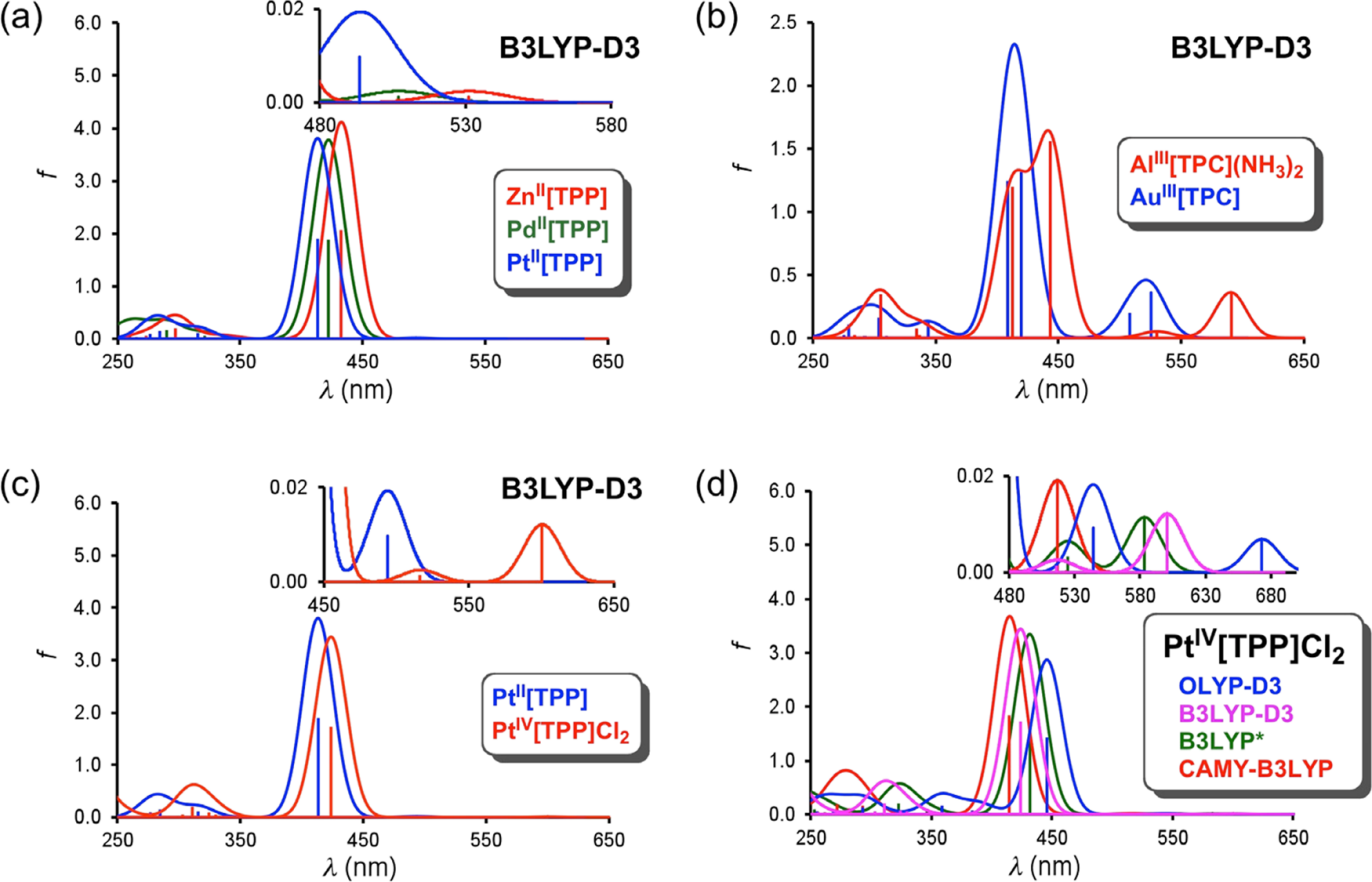
TDDFT (COSMO/dichloromethane) simulated spectra of the metalloporphyrins studied.

We began by examining to what extent TDDFT calculations reproduce known trends in relative positions of the absorption maxima of the compounds studied. As mentioned above, four exchange-correlation functionals were examined—OLYP-D3, B3LYP-D3, B3LYP*, and CAMY-B3LYP—with solvation (dichloromethane) taken into account with the COSMO model. Table 1 lists calculated and experimental absorption maxima and calculated HOMO–LUMO gaps, while Figure 2 presents selected simulated spectra, mostly from B3LYP-D3 calculations. It is immediately obvious that all the exchange-correlation functionals do a qualitatively good job of reproducing key trends in experimental absorption maxima. Thus, both the Q and Soret bands of Pd^II^[TPP] and Pt^II^[TPP] are hypsochromically shifted relative to those of Zn^II^[TPP], with larger blueshifts for Pt, just as experimentally observed.^39−41^ The calculations also predict a substantial spectral blueshift for Au^III^[TPC] relative to Al[TPC](NH_3_)_2_, mirroring a qualitatively similar blueshift for Au^III^[TPFPC] relative to Al^III^[TPFPC](py)_2_.^33,43^ Finally, the calculations predict a spectral redshift for Pt^IV^[TPP]Cl_2_ relative to Pt^II^[TPP], again in qualitative accord with experimental results.^41,42^

**Table 1:** Comparison of TDDFT and Experimental Absorption Maxima (nm)^a^

compound	Q (nm)					Soret (nm)					HOMO–LUMO gap (eV)			
	OLYP	B3LYP	B3LYP*	CAMY-B3LYP	Expt	OLYP	B3LYP	B3LYP*	CAMY-B3LYP	Expt	OLYP	B3LYP	B3LYP*	CAMY-B3LYP
Zn[TPP]	564.3	531.2	542.0	540.4	589	454.3	432.6	441.8	424.8	425	1.94	2.90	2.63	4.42
Pd[TPP]	534.9	507.0	515.6	512.7	554	445.6	422.1	430.7	412.2	418	2.09	3.04	2.78	4.58
Pt[TPP]	519.7	493.7	500.2	494.5	539	437.5	413.2	420.7	400.3	493	2.19	3.14	2.89	4.68
Pt[*TPP*]Cl_2_	673.1	600.3	583.2	523.3	570	445.7	423.8	431.4	414.8	421	1.81	2.87	2.72	4.50
	544.0	516.3	524.5	516.5										
Au[TPC]	554.4	525.9	534.6	530.3	575	449.4	419.4	429.8	408.1	418	1.85	2.75	2.51	4.26
	537.8	508.3	516.7	507.4	560	441.2	408.4	418.5	391.8					
Al[TPC]-(NH_3_)_2_	631.7	590.7	607.6	600.7	620	471.8	443.4	455.9	436.4	432	1.55	2.46	2.20	3.94
	570.9	530.4	544.2	528.1	582	445.4	413.0	424.3	398.0	412				

a The experimental data quoted are obtained from refs (40−44).

Interestingly, the lowest-energy Q band of Pt^IV^[TPP]Cl_2_ appears to pose a peculiar challenge for some of the functionals. Thus, the calculated lowest-energy transition for this compound (experimental value:570 nm^42^) is not a true Q band but a HOMO(a_2u_) → LUMO(a_1g_) transition, where the a_1g_ LUMO corresponds to the empty d_z2_ orbital of the Pt(IV) center. Table 1 shows that while OLYP unduly redshifts this feature, CAMY-B3LYP results in an undue blueshift, whereas B3LYP-D3 and B3LYP* perform just about right.

### 3.2 MO Analysis

**Figure 3:**
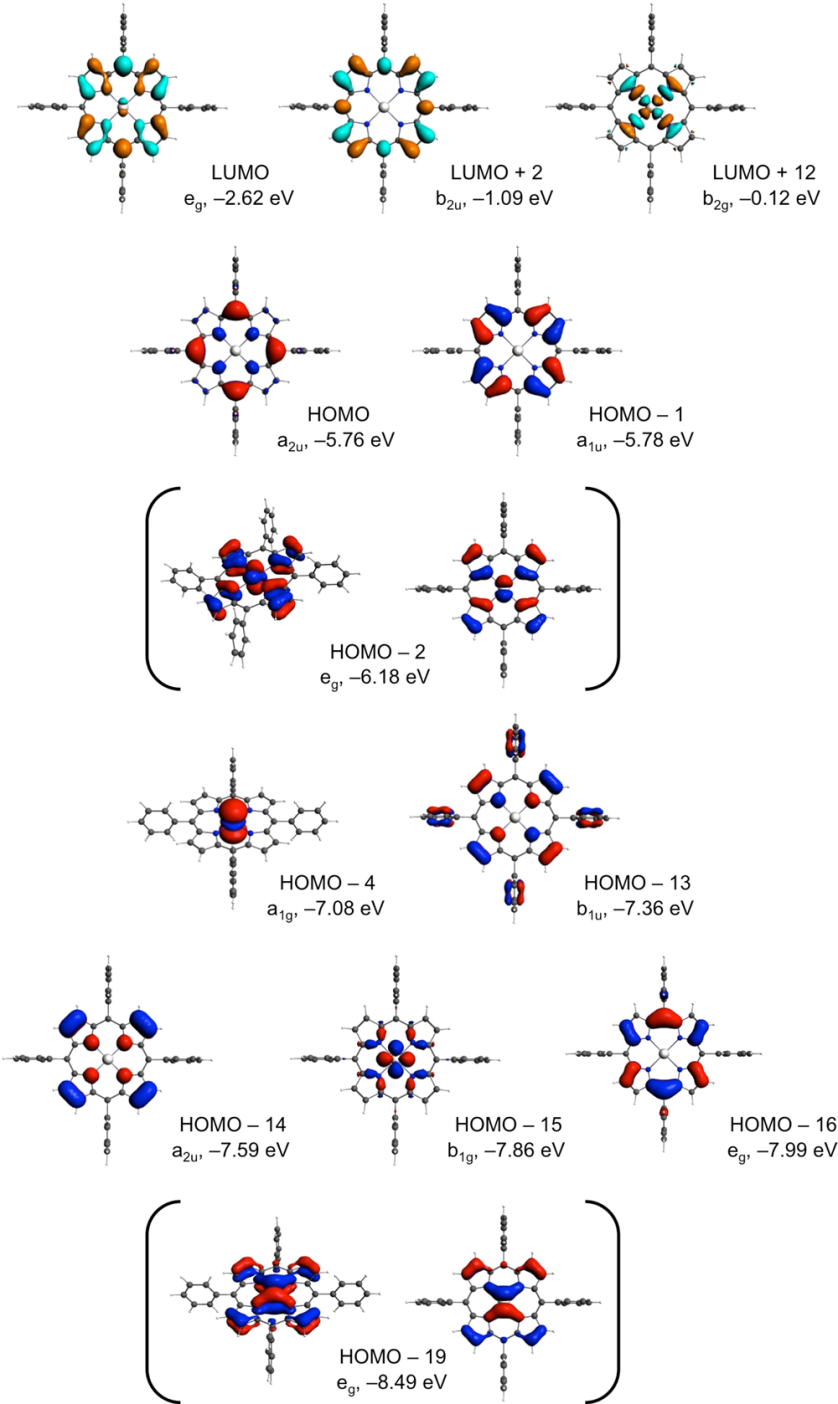
Selected B3LYP-D3 frontier MOs of Pt^II^[TPP].

**Figure 4:**
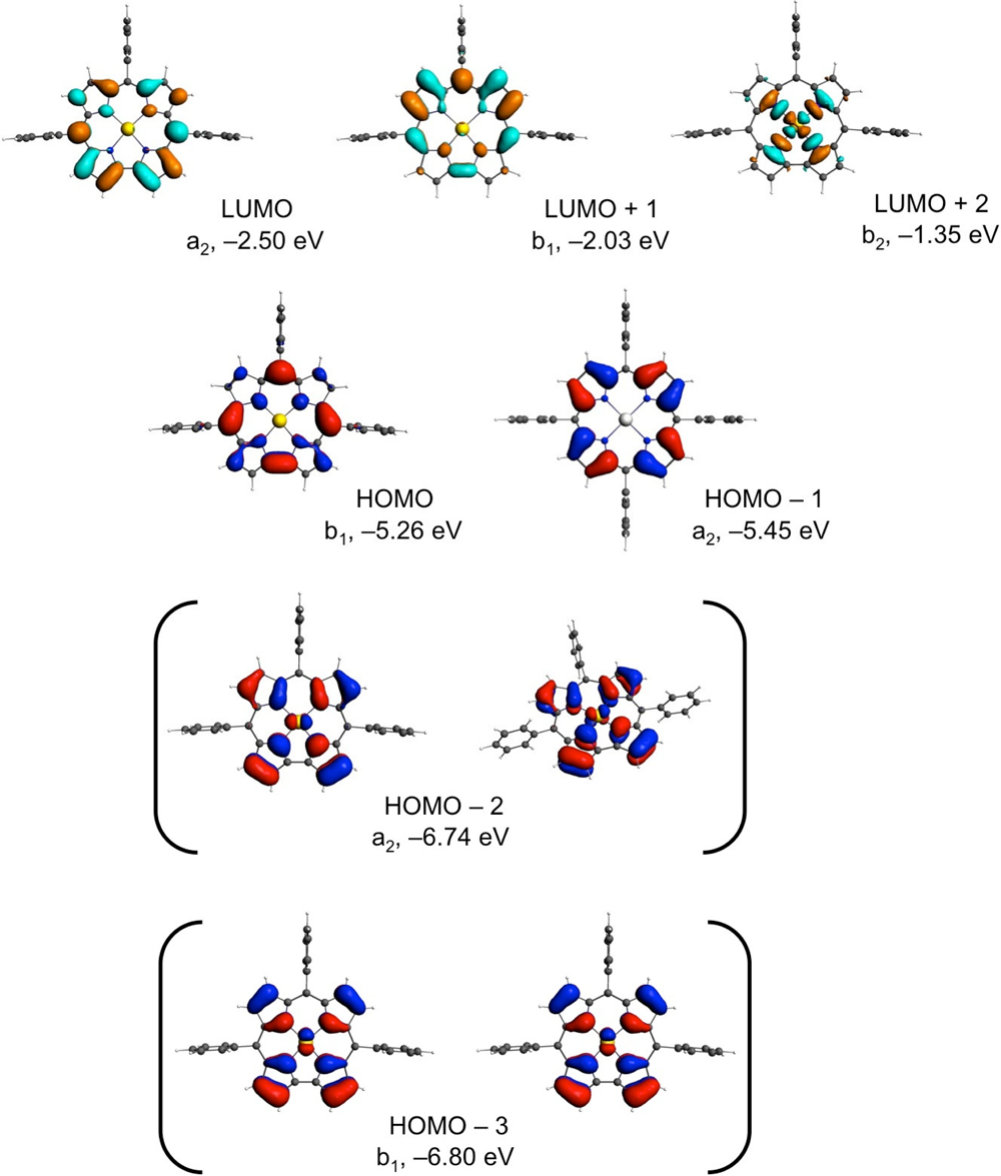
Selected B3LYP-D3 frontier MOs of Au^III^[TPC].

A first step toward understanding the hypsoporphyrin effect is to examine the MO composition of the various calculated spectral features. This information is provided in Table 2 for the B3LYP-D3 functional, while key MOs are depicted in Figure 3 for one of the complexes, Pt^II^[TPP]. To our considerable surprise, we found that the four frontier MOs of all the complexes examined, except Pt^IV^[TPP]Cl_2_, correspond to classic Gouterman MOs, with little or no metal d character. Even for the two corrole derivatives, the four frontier MOs for Au^III^[TPC] (Figure 4) and Al^III^[TPC](NH_3_)_2_ look essentially identical. This finding, reminiscent of the Sherlock Holmes story (*Silver Blaze*) about “the dog that didn’t bark in the night-time”, flies in the face of—and indeed invalidates—the conventional explanation for the hypsoporphyrin effect, namely, that a π-antibonding interaction with the metal d_π_ orbitals is responsible for an elevation of the orbital energies of the e_g_ LUMOs.

**Table 2:** B3LYP-D3/STO-TZ2P TDDFT Results, Including Transition Energies (*E*) and Wavelengths (*l*), Oscillator Strengths (*f*), MO Compositions, and Symmetries

molecule	peak	*E* (eV)	λ (nm)	*f*	MO composition		weight (%)	state symmetry
					from	to		
Zn[TPP]	Q	2.33	531.2	0.001	HOMO	LUMO	55	E_u_
					HOMO – 1	LUMO	45	E_u_
	Soret	2.87	432.6	2.061	HOMO – 1	LUMO	54	E_u_
Pd[TPP]	Q	2.45	507.0	0.001	HOMO – 1	LUMO	50	E_u_
					HOMO	LUMO	49	E_u_
	Soret	2.94	422.1	1.891	HOMO	LUMO	49	E_u_
					HOMO – 1	LUMO	49	E_u_
Pt[TPP]	Q	2.51	493.7	0.010	HOMO – 1	LUMO	55	E_u_
					HOMO	LUMO	44	E_u_
	Soret	3.00	413.2	1.906	HOMO	LUMO	54	E_u_
					HOMO – 1	LUMO	44	E_u_
Pt[TPP]Cl_2_	Q	2.07	600.3	0.012	HOMO	LUMO	100	A_2u_
		2.40	516.3	0.001	HOMO	LUMO + 1	55	E_u_
					HOMO – 1	LUMO + 1	44	E_u_
	Soret	2.93	423.8	1.722	HOMO – 1	LUMO + 1	54	E_u_
					HOMO	LUMO + 1	42	E_u_
Au[TPC]	Q	2.36	525.9	0.362	HOMO	LUMO	88	B_2_
					HOMO – 1	LUMO + 1	11	B_2_
		2.44	508.3	0.204	HOMO – 1	LUMO	81	A_1_
					HOMO	LUMO + 1	18	A_1_
	Soret	2.96	419.4	1.312	HOMO	LUMO + 1	80	A_1_
					HOMO – 1	LUMO	17	A_1_
		3.04	408.4	1.248	HOMO – 1	LUMO + 1	87	B_2_
					HOMO	LUMO	11	B_2_
Al[TPC](NH_3_)_2_	Q	2.10	590.7	0.362	HOMO	LUMO	91	B_2_
					HOMO – 1	LUMO + 1	8	B_2_
		2.34	530.4	0.052	HOMO – 1	LUMO	60	A_1_
					HOMO	LUMO + 1	39	A_1_
	Soret	2.80	443.4	1.561	HOMO	LUMO + 1	59	A_1_
					HOMO – 1	LUMO	39	A_1_
		3.00	413.0	1.204	HOMO – 1	LUMO + 1	91	B_2_
					HOMO	LUMO	7	B_2_

**Figure 5:**
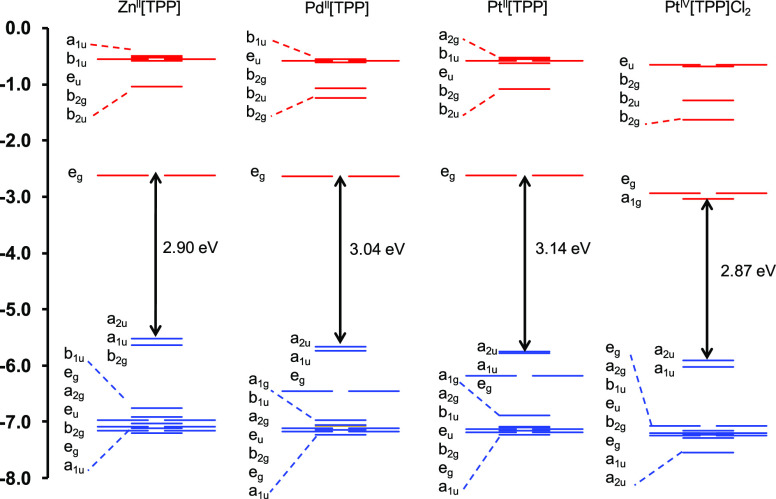
B3LYP-D3 MO energy level diagrams for key TPP derivatives studied.

**Figure 6:**
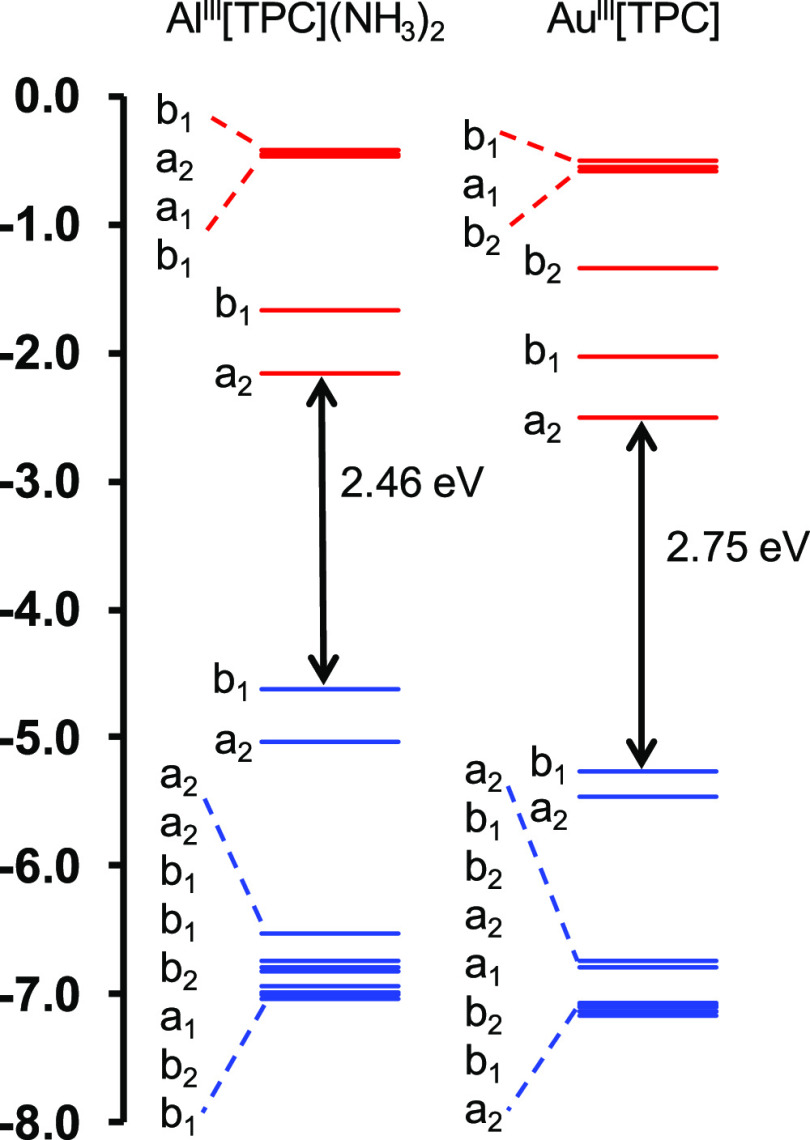
B3LYP-D3 MO energy level diagrams for key TPC derivatives.

A comparative study of the frontier MO energy levels (Figures 5 and 6) came to our rescue. While the LUMO energy levels were found to be almost identical across all the porphyrin (or corrole) derivatives studied, the hypsoporphyrins examined exhibit lower orbital energies for the a_2u_ HOMOs (or for the topologically similar b_1_ HOMOs of corroles). This, then, appears to be the new explanation for the hypsoporphyrin effect.

**Table 3:** Selected Mulliken and Hirschfeld Charges and N 1s Orbital Energies (eV) for the Compounds Studied

		Mulliken		Hirschfield		N 1s energy	
		OLYP	B3LYP-D3	OLYP	B3LYP-D3	OLYP	B3LYP-D3
Zn^II^[TPP]	M	0.815	0.817	0.479	0.481		
	ligand	–0.815	–0.817	–0.479	–0.481		
	N	–0.517	–0.519	–0.160	–0.170	–381.668	–390.126
	C*α*	0.228	0.228	0.024	0.029		
	C*β*	0.236	0.190	–0.068	–0.067		
	C*m*	–0.020	–0.034	–0.005	–0.004		
Pd^II^[TPP]	M	0.991	0.901	0.437	0.472		
	ligand	–0.991	–0.901	–0.437	–0.472		
	N	–0.550	–0.547	–0.158	–0.174	–382.396	–390.790
	C*α*	0.221	0.227	0.019	0.024		
	C*β*	0.241	0.196	–0.066	–0.065		
	C*m*	–0.016	–0.032	–0.007	–0.006		
Pt^II^[TPP]	M	0.975	0.933	0.249	0.263		
	ligand	–0.975	–0.933	–0.249	–0.263		
	N	–0.582	–0.571	–0.124	–0.138	–382.644	–391.036
	C_α_	0.228	0.226	0.022	0.028		
	C_β_	0.247	0.200	–0.065	–0.064		
	C_m_	–0.014	–0.030	–0.005	–0.004		
Pt^IV^[TPP]Cl_2_	M	1.040	1.075	0.510	0.555		
	ligand	–0.355	–0.344	0.072	0.033		
	N	–0.515	–0.513	–0.113	–0.132	–383.100	–391.404
	C*α*	0.250	0.244	0.030	0.035		
	C*β*	0.262	0.216	–0.054	–0.053		
	C*m*	–0.016	–0.029	0.000	0.000		
Au^III^[TPC]	M	1.461	1.362	0.551	0.583		
	ligand	–1.461	–1.362	–0.551	–0.583		
	N2	–0.629	–0.607	–0.130	–0.142	–382.740	–391.090
	N1	–0.594	–0.573	–0.136	–0.150	–382.663	–391.089
	C*α*	0.209	0.200	0.012	0.016		
	C*β*	0.219	0.182	–0.072	–0.072		
	C*m*	–0.056	–0.068	–0.017	–0.017		
Al^III^[TPC](NH_3_)_2_	M	1.660	1.640	0.390	0.424		
	ligand	–1.826	–1.817	–0.929	–0.938		
	N2	–0.635	–0.634	–0.155	–0.170	–381.014	–389.439
	N1	–0.548	–0.559	–0.159	–0.175	–381.191	–389.621
	N (NH_3_)	0.237	0.163	–0.167	–0.186	–381.875	–390.341
	C*α*	0.203	0.192	0.016	0.022		
	C*β*	0.196	0.157	–0.087	–0.086		
	C*m*	–0.074	–0.072	–0.022	–0.020		

### 3.3 Molecular Charge Distributions

The question as to why hypsoporphyrins such as Pd^II^[TPP] and Pt^II^[TPP], as well as hypsocorroles such as Au^III^[TPC], should exhibit lower “a_2u_” energy levels is a somewhat subtle one, because, as mentioned, there is little difference in the shape of these orbitals relative to those of the normal porphyrin Zn^II^[TPP] [and the normal corrole Al^III^[TPC](NH_3_)_2_]. An examination of the atomic Mulliken and Hirschfeld charges, as well as of the nitrogen 1s orbital energies (Table 3), suggests a plausible explanation. Hypsoporphyrins appear to involve less electropositive metals that transfer less electron density to the porphyrin/corrole ligands as a whole and specifically to the macrocycle nitrogens. Thus, both the macrocyclic ligands as a whole and their central nitrogens carry less negative Hirschfeld charges in the case of the hypsoporphyrins, relative to the normal porphyrin Zn^II^[TPP]. As a result, the nitrogen 1s orbital energies are also relatively more negative, which would translate to higher XPS ionization potentials, for the hypsoporphyrins. Given that the a_2u_ HOMO has large amplitudes on the macrocycle nitrogens, it follows that hypsoporphyrins should also exhibit lower a_2u_ orbital energies, which accounts for the hypsoporphyrin effect.

The above argument might suggest that a Pt(IV) porphyrin would exhibit a stronger hypsochromic shift than a Pt(II) porphyrin. As shown in Table 1, the opposite is observed. A recent, combined X-ray absorption spectroscopy and density functional theory (DFT) study has shown that a Pt(IV) porphyrin entails substantial oxidation of the porphyrin ligand as a whole.^54^ That systemic oxidation results in a lowering of not only the a_2u_ HOMO, but also an even greater lowering of the e_g_LUMOs, which explains the lack of a hypsoporphyrin spectrum for Pt^IV^[TPP]Cl_2_.

## 4 Conclusions

DFT and TDDFT calculations indicate that the hypsoporphyrin effect (blueshifted Q and Soret bands) does not result from elevated porphyrin LUMO (e_g_) energy levels as a result of antibonding interactions with metal d_π_ orbitals. Instead the observed blueshifts reflect a lowering of the a_2u_ HOMO energy level. Electronegative metals such as Pd and Pt transfer smaller quantities of electron density to the porphyrin nitrogens, compared to a more electropositive metal such as Zn. As a result, the nitrogens in Pd and Pt porphyrins exhibit higher electrostatic potentials, more negative N 1s orbital energies, and higher N 1s ionization potentials. With large amplitudes at the porphyrin nitrogens, the a_2u_ HOMOs of Pd(II) and Pt(II) porphyrins also exhibit lower orbital energies (mirroring the behavior of the N 1s orbitals) than Zn(II) porphyrins, thus explaining the hypsoporphyrin spectra.

The hypsoporphyrin concept also appears to extend to corroles. With blueshifted spectral features relative to six-coordinate Al(III) corroles, Au(III) corroles appear to be justifiably described as hypsocorroles. It may be recalled that examples hypercorroles, likewise, have also been documented in the literature.^55^

## Supporting Information Available

Optimized Cartesian coordinates (PDF)
