# Supplementary material for: The Dog That Didn’t Bark: A New Interpretation of Hypsoporphyrin Spectra and the Question of Hypsocorroles
Source: J Phys Chem A. 2021 Nov 11;125(46):9962–8. doi: 10.1021/acs.jpca.1c08425 (PMC8630793; doi:10.1021/acs.jpca.1c08425)
Supplement: Supplementary file 1 — jp1c08425_si_001.pdf [file jp1c08425_si_001.pdf]

Supporting Information

The Dog That Didn't Bark: A New Interpretation of Hypsoporphyrin Spectra and the Question of Hypsocorroles

Abhik Ghosh*[,a] and Jeanet Conradie,*[,a,b]

[a] Department of Chemistry, UiT – The Arctic University of Norway, N-9037 Tromsø, Norway;

[b]Department of Chemistry, University of the Free State, P.O. Box 339, Bloemfontein 9300, Republic of South Africa.

All-electron B3LYP-D3/ZORA-STO-TZ2P optimized Cartesian coordinates (Å)

Table of Contents ....
1. Zn[TPP] S2
2. Pd[TPP] S3
3. Pt[TPP] S5
4. Pt[TPP]Cl_2 .... S6
5. H_2[TPP] S8
6. Au[TPC] S10
7. Al[TPC]$(NH_3)_2$ S11

1. Zn[TPP]

C	0.000000000	3.447235000	0.000000000
C	0.000000000	4.943823000	0.000000000
C	0.000000000	5.651352000	1.202024000
C	0.000000000	5.651352000	-1.202024000
C	0.000000000	7.042037000	1.202776000
C	0.000000000	7.042037000	-1.202776000
C	0.000000000	7.740535000	0.000000000
C	0.000000000	-3.447235000	0.000000000
C	0.000000000	-4.943823000	0.000000000
C	0.000000000	-5.651352000	1.202024000
C	0.000000000	-5.651352000	-1.202024000
C	0.000000000	-7.042037000	1.202776000
C	0.000000000	-7.042037000	-1.202776000
C	0.000000000	-7.740535000	0.000000000
C	1.243281000	2.804355000	0.000000000
C	1.243281000	-2.804355000	0.000000000
C	2.518687000	3.478124000	0.000000000
C	2.518687000	-3.478124000	0.000000000
C	2.804355000	1.243281000	0.000000000
C	2.804355000	-1.243281000	0.000000000
C	3.447235000	0.000000000	0.000000000
C	3.478124000	2.518687000	0.000000000
C	3.478124000	-2.518687000	0.000000000
C	4.943823000	0.000000000	0.000000000
C	5.651352000	0.000000000	1.202024000
C	5.651352000	0.000000000	-1.202024000
C	7.042037000	0.000000000	1.202776000
C	7.042037000	0.000000000	-1.202776000
C	7.740535000	0.000000000	0.000000000
C	-1.243281000	2.804355000	0.000000000
C	-1.243281000	-2.804355000	0.000000000
C	-2.518687000	3.478124000	0.000000000
C	-2.518687000	-3.478124000	0.000000000
C	-2.804355000	1.243281000	0.000000000
C	-2.804355000	-1.243281000	0.000000000
C	-3.447235000	0.000000000	0.000000000
C	-3.478124000	2.518687000	0.000000000
C	-3.478124000	-2.518687000	0.000000000
C	-4.943823000	0.000000000	0.000000000
C	-5.651352000	0.000000000	1.202024000
C	-5.651352000	0.000000000	-1.202024000
C	-7.042037000	0.000000000	1.202776000
C	-7.042037000	0.000000000	-1.202776000
C	-7.740535000	0.000000000	0.000000000
H	0.000000000	5.109379000	2.138205000
H	0.000000000	5.109379000	-2.138205000
H	0.000000000	7.578377000	2.142217000
H	0.000000000	7.578377000	-2.142217000

H	0.000000000	8.822230000	0.000000000
H	0.000000000	-5.109379000	2.138205000
H	0.000000000	-5.109379000	-2.138205000
H	0.000000000	-7.578377000	2.142217000
H	0.000000000	-7.578377000	-2.142217000
H	0.000000000	-8.822230000	0.000000000
H	2.656243000	4.545732000	0.000000000
H	2.656243000	-4.545732000	0.000000000
H	4.545732000	2.656243000	0.000000000
H	4.545732000	-2.656243000	0.000000000
H	5.109379000	0.000000000	2.138205000
H	5.109379000	0.000000000	-2.138205000
H	7.578377000	0.000000000	2.142217000
H	7.578377000	0.000000000	-2.142217000
H	8.822230000	0.000000000	0.000000000
H	-2.656243000	4.545732000	0.000000000
H	-2.656243000	-4.545732000	0.000000000
H	-4.545732000	2.656243000	0.000000000
H	-4.545732000	-2.656243000	0.000000000
H	-5.109379000	0.000000000	2.138205000
H	-5.109379000	0.000000000	-2.138205000
H	-7.578377000	0.000000000	2.142217000
H	-7.578377000	0.000000000	-2.142217000
H	-8.822230000	0.000000000	0.000000000
N	1.448412000	1.448412000	0.000000000
N	1.448412000	-1.448412000	0.000000000
N	-1.448412000	1.448412000	0.000000000
N	-1.448412000	-1.448412000	0.000000000
Zn	0.000000000	0.000000000	0.000000000

2. Pd[TPP]

C	0.000000000	3.442247000	0.000000000
C	0.000000000	4.937353000	0.000000000
C	0.000000000	5.643027000	1.202618000
C	0.000000000	5.643027000	-1.202618000
C	0.000000000	7.033586000	1.203083000
C	0.000000000	7.033586000	-1.203083000
C	0.000000000	7.731624000	0.000000000
C	0.000000000	-3.442247000	0.000000000
C	0.000000000	-4.937353000	0.000000000
C	0.000000000	-5.643027000	1.202618000
C	0.000000000	-5.643027000	-1.202618000
C	0.000000000	-7.033586000	1.203083000
C	0.000000000	-7.033586000	-1.203083000
C	0.000000000	-7.731624000	0.000000000
C	1.232674000	2.794308000	0.000000000
C	1.232674000	-2.794308000	0.000000000
C	2.507182000	3.464529000	0.000000000
C	2.507182000	-3.464529000	0.000000000

C	2.794308000	1.232674000	0.000000000
C	2.794308000	-1.232674000	0.000000000
C	3.442247000	0.000000000	0.000000000
C	3.464529000	2.507182000	0.000000000
C	3.464529000	-2.507182000	0.000000000
C	4.937353000	0.000000000	0.000000000
C	5.643027000	0.000000000	1.202618000
C	5.643027000	0.000000000	-1.202618000
C	7.033586000	0.000000000	1.203083000
C	7.033586000	0.000000000	-1.203083000
C	7.731624000	0.000000000	0.000000000
C	-1.232674000	2.794308000	0.000000000
C	-1.232674000	-2.794308000	0.000000000
C	-2.507182000	3.464529000	0.000000000
C	-2.507182000	-3.464529000	0.000000000
C	-2.794308000	1.232674000	0.000000000
C	-2.794308000	-1.232674000	0.000000000
C	-3.442247000	0.000000000	0.000000000
C	-3.464529000	2.507182000	0.000000000
C	-3.464529000	-2.507182000	0.000000000
C	-4.937353000	0.000000000	0.000000000
C	-5.643027000	0.000000000	1.202618000
C	-5.643027000	0.000000000	-1.202618000
C	-7.033586000	0.000000000	1.203083000
C	-7.033586000	0.000000000	-1.203083000
C	-7.731624000	0.000000000	0.000000000
H	0.000000000	5.100477000	2.138428000
H	0.000000000	5.100477000	-2.138428000
H	0.000000000	7.570183000	2.142403000
H	0.000000000	7.570183000	-2.142403000
H	0.000000000	8.813453000	0.000000000
H	0.000000000	-5.100477000	2.138428000
H	0.000000000	-5.100477000	-2.138428000
H	0.000000000	-7.570183000	2.142403000
H	0.000000000	-7.570183000	-2.142403000
H	0.000000000	-8.813453000	0.000000000
H	2.639483000	4.532455000	0.000000000
H	2.639483000	-4.532455000	0.000000000
H	4.532455000	2.639483000	0.000000000
H	4.532455000	-2.639483000	0.000000000
H	5.100477000	0.000000000	2.138428000
H	5.100477000	0.000000000	-2.138428000
H	7.570183000	0.000000000	2.142403000
H	7.570183000	0.000000000	-2.142403000
H	8.813453000	0.000000000	0.000000000
H	-2.639483000	4.532455000	0.000000000
H	-2.639483000	-4.532455000	0.000000000
H	-4.532455000	2.639483000	0.000000000
H	-4.532455000	-2.639483000	0.000000000
H	-5.100477000	0.000000000	2.138428000
H	-5.100477000	0.000000000	-2.138428000

H	-7.570183000	0.000000000	2.142403000
H	-7.570183000	0.000000000	-2.142403000
H	-8.813453000	0.000000000	0.000000000
N	1.437576000	1.437576000	0.000000000
N	1.437576000	-1.437576000	0.000000000
N	-1.437576000	1.437576000	0.000000000
N	-1.437576000	-1.437576000	0.000000000
Pd	0.000000000	0.000000000	0.000000000

3. Pt[TPP]

C	0.000000000	3.442030000	0.000000000
C	0.000000000	4.936758000	0.000000000
C	0.000000000	5.641687000	1.202228000
C	0.000000000	5.641687000	-1.202228000
C	0.000000000	7.031998000	1.202832000
C	0.000000000	7.031998000	-1.202832000
C	0.000000000	7.729923000	0.000000000
C	0.000000000	-3.442030000	0.000000000
C	0.000000000	-4.936758000	0.000000000
C	0.000000000	-5.641687000	1.202228000
C	0.000000000	-5.641687000	-1.202228000
C	0.000000000	-7.031998000	1.202832000
C	0.000000000	-7.031998000	-1.202832000
C	0.000000000	-7.729923000	0.000000000
C	1.231668000	2.796468000	0.000000000
C	1.231668000	-2.796468000	0.000000000
C	2.506117000	3.463222000	0.000000000
C	2.506117000	-3.463222000	0.000000000
C	2.796468000	1.231668000	0.000000000
C	2.796468000	-1.231668000	0.000000000
C	3.442030000	0.000000000	0.000000000
C	3.463222000	2.506117000	0.000000000
C	3.463222000	-2.506117000	0.000000000
C	4.936758000	0.000000000	0.000000000
C	5.641687000	0.000000000	1.202228000
C	5.641687000	0.000000000	-1.202228000
C	7.031998000	0.000000000	1.202832000
C	7.031998000	0.000000000	-1.202832000
C	7.729923000	0.000000000	0.000000000
C	-1.231668000	2.796468000	0.000000000
C	-1.231668000	-2.796468000	0.000000000
C	-2.506117000	3.463222000	0.000000000
C	-2.506117000	-3.463222000	0.000000000
C	-2.796468000	1.231668000	0.000000000
C	-2.796468000	-1.231668000	0.000000000
C	-3.442030000	0.000000000	0.000000000
C	-3.463222000	2.506117000	0.000000000
C	-3.463222000	-2.506117000	0.000000000
C	-4.936758000	0.000000000	0.000000000

C	-5.641687000	0.000000000	1.202228000
C	-5.641687000	0.000000000	-1.202228000
C	-7.031998000	0.000000000	1.202832000
C	-7.031998000	0.000000000	-1.202832000
C	-7.729923000	0.000000000	0.000000000
H	0.000000000	5.098369000	2.137826000
H	0.000000000	5.098369000	-2.137826000
H	0.000000000	7.568737000	2.142282000
H	0.000000000	7.568737000	-2.142282000
H	0.000000000	8.811865000	0.000000000
H	0.000000000	-5.098369000	2.137826000
H	0.000000000	-5.098369000	-2.137826000
H	0.000000000	-7.568737000	2.142282000
H	0.000000000	-7.568737000	-2.142282000
H	0.000000000	-8.811865000	0.000000000
H	2.638439000	4.531114000	0.000000000
H	2.638439000	-4.531114000	0.000000000
H	4.531114000	2.638439000	0.000000000
H	4.531114000	-2.638439000	0.000000000
H	5.098369000	0.000000000	2.137826000
H	5.098369000	0.000000000	-2.137826000
H	7.568737000	0.000000000	2.142282000
H	7.568737000	0.000000000	-2.142282000
H	8.811865000	0.000000000	0.000000000
H	-2.638439000	4.531114000	0.000000000
H	-2.638439000	-4.531114000	0.000000000
H	-4.531114000	2.638439000	0.000000000
H	-4.531114000	-2.638439000	0.000000000
H	-5.098369000	0.000000000	2.137826000
H	-5.098369000	0.000000000	-2.137826000
H	-7.568737000	0.000000000	2.142282000
H	-7.568737000	0.000000000	-2.142282000
H	-8.811865000	0.000000000	0.000000000
N	1.435304000	1.435304000	0.000000000
N	1.435304000	-1.435304000	0.000000000
N	-1.435304000	1.435304000	0.000000000
N	-1.435304000	-1.435304000	0.000000000
Pt	0.000000000	0.000000000	0.000000000

4. Pt[TPP]Cl$_2$

C	0.000000000	3.446677000	0.000000000
C	0.000000000	4.941006000	0.000000000
C	0.000000000	5.644346000	1.203283000
C	0.000000000	5.644346000	-1.203283000
C	0.000000000	7.034518000	1.203254000
C	0.000000000	7.034518000	-1.203254000
C	0.000000000	7.731861000	0.000000000
C	0.000000000	-3.446677000	0.000000000
C	0.000000000	-4.941006000	0.000000000

C	0.000000000	-5.644346000	1.203283000
C	0.000000000	-5.644346000	-1.203283000
C	0.000000000	-7.034518000	1.203254000
C	0.000000000	-7.034518000	-1.203254000
C	0.000000000	-7.731861000	0.000000000
C	1.231082000	2.799965000	0.000000000
C	1.231082000	-2.799965000	0.000000000
C	2.505408000	3.463021000	0.000000000
C	2.505408000	-3.463021000	0.000000000
C	2.799965000	1.231082000	0.000000000
C	2.799965000	-1.231082000	0.000000000
C	3.446677000	0.000000000	0.000000000
C	3.463021000	2.505408000	0.000000000
C	3.463021000	-2.505408000	0.000000000
C	4.941006000	0.000000000	0.000000000
C	5.644346000	0.000000000	1.203283000
C	5.644346000	0.000000000	-1.203283000
C	7.034518000	0.000000000	1.203254000
C	7.034518000	0.000000000	-1.203254000
C	7.731861000	0.000000000	0.000000000
C	-1.231082000	2.799965000	0.000000000
C	-1.231082000	-2.799965000	0.000000000
C	-2.505408000	3.463021000	0.000000000
C	-2.505408000	-3.463021000	0.000000000
C	-2.799965000	1.231082000	0.000000000
C	-2.799965000	-1.231082000	0.000000000
C	-3.446677000	0.000000000	0.000000000
C	-3.463021000	2.505408000	0.000000000
C	-3.463021000	-2.505408000	0.000000000
C	-4.941006000	0.000000000	0.000000000
C	-5.644346000	0.000000000	1.203283000
C	-5.644346000	0.000000000	-1.203283000
C	-7.034518000	0.000000000	1.203254000
C	-7.034518000	0.000000000	-1.203254000
C	-7.731861000	0.000000000	0.000000000
Cl	0.000000000	0.000000000	2.361558000
Cl	0.000000000	0.000000000	-2.361558000
H	0.000000000	5.100861000	2.138546000
H	0.000000000	5.100861000	-2.138546000
H	0.000000000	7.571282000	2.142427000
H	0.000000000	7.571282000	-2.142427000
H	0.000000000	8.813695000	0.000000000
H	0.000000000	-5.100861000	2.138546000
H	0.000000000	-5.100861000	-2.138546000
H	0.000000000	-7.571282000	2.142427000
H	0.000000000	-7.571282000	-2.142427000
H	0.000000000	-8.813695000	0.000000000
H	2.636761000	4.530631000	0.000000000
H	2.636761000	-4.530631000	0.000000000
H	4.530631000	2.636761000	0.000000000
H	4.530631000	-2.636761000	0.000000000

H	5.100861000	0.000000000	2.138546000
H	5.100861000	0.000000000	-2.138546000
H	7.571282000	0.000000000	2.142427000
H	7.571282000	0.000000000	-2.142427000
H	8.813695000	0.000000000	0.000000000
H	-2.636761000	4.530631000	0.000000000
H	-2.636761000	-4.530631000	0.000000000
H	-4.530631000	2.636761000	0.000000000
H	-4.530631000	-2.636761000	0.000000000
H	-5.100861000	0.000000000	2.138546000
H	-5.100861000	0.000000000	-2.138546000
H	-7.571282000	0.000000000	2.142427000
H	-7.571282000	0.000000000	-2.142427000
H	-8.813695000	0.000000000	0.000000000
N	1.445204000	1.445204000	0.000000000
N	1.445204000	-1.445204000	0.000000000
N	-1.445204000	1.445204000	0.000000000
N	-1.445204000	-1.445204000	0.000000000
Pt	0.000000000	0.000000000	0.000000000

5. H_2[TPP]

C	0.675175000	4.242859000	0.153219000
C	0.675175000	-4.242859000	0.153219000
C	1.089131000	2.855975000	0.012086000
C	1.089131000	-2.855975000	0.012086000
C	2.434073000	2.452589000	-0.026186000
C	2.434073000	-2.452589000	-0.026186000
C	2.879445000	1.128676000	-0.070635000
C	2.879445000	-1.128676000	-0.070635000
C	3.487148000	3.510995000	-0.008526000
C	3.487148000	-3.510995000	-0.008526000
C	3.660442000	4.360689000	-1.102351000
C	3.660442000	-4.360689000	-1.102351000
C	4.233465000	0.681923000	-0.170038000
C	4.233465000	-0.681923000	-0.170038000
C	4.315787000	3.668512000	1.103983000
C	4.315787000	-3.668512000	1.103983000
C	4.641203000	5.345033000	-1.085141000
C	4.641203000	-5.345033000	-1.085141000
C	5.293552000	4.656481000	1.124627000
C	5.293552000	-4.656481000	1.124627000
C	5.459656000	5.496669000	0.029125000
C	5.459656000	-5.496669000	0.029125000
C	-0.675175000	4.242859000	0.153219000
C	-0.675175000	-4.242859000	0.153219000
C	-1.089131000	2.855975000	0.012086000
C	-1.089131000	-2.855975000	0.012086000
C	-2.434073000	2.452589000	-0.026186000

C	-2.434073000	-2.452589000	-0.026186000
C	-2.879445000	1.128676000	-0.070635000
C	-2.879445000	-1.128676000	-0.070635000
C	-3.487148000	3.510995000	-0.008526000
C	-3.487148000	-3.510995000	-0.008526000
C	-3.660442000	4.360689000	-1.102351000
C	-3.660442000	-4.360689000	-1.102351000
C	-4.233465000	0.681923000	-0.170038000
C	-4.233465000	-0.681923000	-0.170038000
C	-4.315787000	3.668512000	1.103983000
C	-4.315787000	-3.668512000	1.103983000
C	-4.641203000	5.345033000	-1.085141000
C	-4.641203000	-5.345033000	-1.085141000
C	-5.293552000	4.656481000	1.124627000
C	-5.293552000	-4.656481000	1.124627000
C	-5.459656000	5.496669000	0.029125000
C	-5.459656000	-5.496669000	0.029125000
H	1.091776000	0.000000000	0.014327000
H	1.332479000	5.088955000	0.255019000
H	1.332479000	-5.088955000	0.255019000
H	3.023865000	4.245243000	-1.969464000
H	3.023865000	-4.245243000	-1.969464000
H	4.187435000	3.016219000	1.957586000
H	4.187435000	-3.016219000	1.957586000
H	4.765967000	5.992936000	-1.942825000
H	4.765967000	-5.992936000	-1.942825000
H	5.087399000	1.331354000	-0.241086000
H	5.087399000	-1.331354000	-0.241086000
H	5.923248000	4.769997000	1.997264000
H	5.923248000	-4.769997000	1.997264000
H	6.221689000	6.264801000	0.043581000
H	6.221689000	-6.264801000	0.043581000
H	-1.091776000	0.000000000	0.014327000
H	-1.332479000	5.088955000	0.255019000
H	-1.332479000	-5.088955000	0.255019000
H	-3.023865000	4.245243000	-1.969464000
H	-3.023865000	-4.245243000	-1.969464000
H	-4.187435000	3.016219000	1.957586000
H	-4.187435000	-3.016219000	1.957586000
H	-4.765967000	5.992936000	-1.942825000
H	-4.765967000	-5.992936000	-1.942825000
H	-5.087399000	1.331354000	-0.241086000
H	-5.087399000	-1.331354000	-0.241086000
H	-5.923248000	4.769997000	1.997264000
H	-5.923248000	-4.769997000	1.997264000
H	-6.221689000	6.264801000	0.043581000
H	-6.221689000	-6.264801000	0.043581000
N	0.000000000	2.037539000	-0.059490000
N	0.000000000	-2.037539000	-0.059490000
N	2.100654000	0.000000000	-0.026226000
N	-2.100654000	0.000000000	-0.026226000

6. Au[TPC]

Au	0.000000000	0.000000000	-0.555740000
C	0.000000000	0.000000000	2.771626000
C	0.000000000	0.000000000	4.265173000
C	0.000000000	0.000000000	7.061102000
C	0.000000000	0.715399000	-3.306034000
C	0.000000000	1.253724000	2.130266000
C	0.000000000	1.825419000	-4.192426000
C	0.000000000	2.564401000	2.722291000
C	0.000000000	2.611782000	-2.042352000
C	0.000000000	2.794292000	0.454059000
C	0.000000000	2.983610000	-3.425477000
C	0.000000000	3.353318000	-0.837974000
C	0.000000000	3.488104000	1.713883000
C	0.000000000	4.841517000	-0.947074000
C	0.000000000	7.630886000	-1.142242000
C	0.000000000	-0.715399000	-3.306034000
C	0.000000000	-1.253724000	2.130266000
C	0.000000000	-1.825419000	-4.192426000
C	0.000000000	-2.564401000	2.722291000
C	0.000000000	-2.611782000	-2.042352000
C	0.000000000	-2.794292000	0.454059000
C	0.000000000	-2.983610000	-3.425477000
C	0.000000000	-3.353318000	-0.837974000
C	0.000000000	-3.488104000	1.713883000
C	0.000000000	-4.841517000	-0.947074000
C	0.000000000	-7.630886000	-1.142242000
C	1.201576000	0.000000000	4.972643000
C	1.201831000	5.546947000	-0.998680000
C	1.201831000	-5.546947000	-0.998680000
C	1.202454000	6.934268000	-1.095276000
C	1.202454000	-6.934268000	-1.095276000
C	1.202824000	0.000000000	6.362918000
C	-1.201576000	0.000000000	4.972643000
C	-1.201831000	5.546947000	-0.998680000
C	-1.201831000	-5.546947000	-0.998680000
C	-1.202454000	6.934268000	-1.095276000
C	-1.202454000	-6.934268000	-1.095276000
C	-1.202824000	0.000000000	6.362918000
H	0.000000000	0.000000000	8.143217000
H	0.000000000	1.771631000	-5.268550000
H	0.000000000	2.757751000	3.781517000
H	0.000000000	3.995969000	-3.793330000
H	0.000000000	4.560109000	1.817585000
H	0.000000000	8.710350000	-1.215793000
H	0.000000000	-1.771631000	-5.268550000
H	0.000000000	-2.757751000	3.781517000
H	0.000000000	-3.995969000	-3.793330000
H	0.000000000	-4.560109000	1.817585000
H	0.000000000	-8.710350000	-1.215793000

H	2.137246000	0.000000000	4.429025000
H	2.137563000	5.004800000	-0.960727000
H	2.137563000	-5.004800000	-0.960727000
H	2.141798000	7.470234000	-1.132794000
H	2.141798000	-7.470234000	-1.132794000
H	2.142354000	0.000000000	6.899818000
H	-2.137246000	0.000000000	4.429025000
H	-2.137563000	5.004800000	-0.960727000
H	-2.137563000	-5.004800000	-0.960727000
H	-2.141798000	7.470234000	-1.132794000
H	-2.141798000	-7.470234000	-1.132794000
H	-2.142354000	0.000000000	6.899818000
N	0.000000000	1.251748000	-2.045061000
N	0.000000000	1.446880000	0.773617000
N	0.000000000	-1.251748000	-2.045061000
N	0.000000000	-1.446880000	0.773617000

7. Al[TPC]$(NH_3)_2$

Al	0.000000000	0.000000000	-0.652933000
C	0.000000000	0.000000000	2.627168000
C	0.000000000	0.000000000	4.120109000
C	0.000000000	0.000000000	6.922550000
C	0.000000000	0.717164000	-3.355887000
C	0.000000000	1.248861000	1.975265000
C	0.000000000	1.796946000	-4.277701000
C	0.000000000	2.550177000	2.586210000
C	0.000000000	2.596351000	-2.147095000
C	0.000000000	2.780262000	0.328181000
C	0.000000000	2.968272000	-3.527596000
C	0.000000000	3.355658000	-0.952932000
C	0.000000000	3.479343000	1.582990000
C	0.000000000	4.841935000	-1.067998000
C	0.000000000	7.633980000	-1.291134000
C	0.000000000	-0.717164000	-3.355887000
C	0.000000000	-1.248861000	1.975265000
C	0.000000000	-1.796946000	-4.277701000
C	0.000000000	-2.550177000	2.586210000
C	0.000000000	-2.596351000	-2.147095000
C	0.000000000	-2.780262000	0.328181000
C	0.000000000	-2.968272000	-3.527596000
C	0.000000000	-3.355658000	-0.952932000
C	0.000000000	-3.479343000	1.582990000
C	0.000000000	-4.841935000	-1.067998000
C	0.000000000	-7.633980000	-1.291134000
C	1.199746000	0.000000000	4.832516000
C	1.200516000	5.550421000	-1.127274000
C	1.200516000	-5.550421000	-1.127274000
C	1.202142000	0.000000000	6.223259000
C	1.202274000	6.936920000	-1.237242000

C	1.202274000	-6.936920000	-1.237242000
C	-1.199746000	0.000000000	4.832516000
C	-1.200516000	5.550421000	-1.127274000
C	-1.200516000	-5.550421000	-1.127274000
C	-1.202142000	0.000000000	6.223259000
C	-1.202274000	6.936920000	-1.237242000
C	-1.202274000	-6.936920000	-1.237242000
H	0.000000000	0.000000000	8.004772000
H	0.000000000	1.720454000	-5.353123000
H	0.000000000	2.736236000	3.647236000
H	0.000000000	3.977189000	-3.907248000
H	0.000000000	4.551439000	1.689971000
H	0.000000000	8.712909000	-1.374649000
H	0.000000000	-1.720454000	-5.353123000
H	0.000000000	-2.736236000	3.647236000
H	0.000000000	-3.977189000	-3.907248000
H	0.000000000	-4.551439000	1.689971000
H	0.000000000	-8.712909000	-1.374649000
H	2.135975000	0.000000000	4.289419000
H	2.136691000	5.009174000	-1.083691000
H	2.136691000	-5.009174000	-1.083691000
H	2.142085000	0.000000000	6.760020000
H	2.142146000	7.471911000	-1.278986000
H	2.142146000	-7.471911000	-1.278986000
H	2.509824000	0.000000000	-1.633515000
H	2.538979000	0.816879000	-0.218849000
H	2.538979000	-0.816879000	-0.218849000
H	-2.135975000	0.000000000	4.289419000
H	-2.136691000	5.009174000	-1.083691000
H	-2.136691000	-5.009174000	-1.083691000
H	-2.142085000	0.000000000	6.760020000
H	-2.142146000	7.471911000	-1.278986000
H	-2.142146000	-7.471911000	-1.278986000
H	-2.509824000	0.000000000	-1.633515000
H	-2.538979000	0.816879000	-0.218849000
H	-2.538979000	-0.816879000	-0.218849000
N	0.000000000	1.239361000	-2.097695000
N	0.000000000	1.420078000	0.614653000
N	0.000000000	-1.239361000	-2.097695000
N	0.000000000	-1.420078000	0.614653000
N	2.154524000	0.000000000	-0.682859000
N	-2.154524000	0.000000000	-0.682859000
